# Roles of sigma-1 receptors on mitochondrial functions relevant to neurodegenerative diseases

**DOI:** 10.1186/s12929-017-0380-6

**Published:** 2017-09-16

**Authors:** Tzu-Yu Weng, Shang-Yi Anne Tsai, Tsung-Ping Su

**Affiliations:** 1 Cellular Pathobiology Section, Integrative Neuroscience Branch, Intramural Research Program, National Institute on Drug Abuse, NIH, DHHS, IRP, NIDA/NIH, Triad Bldg. suite 3512, 333 Cassell Drive, Baltimore, MD 21224 USA; 2 Genomics Research Center, Academia Sinica, Taipei, Taiwan

**Keywords:** Sigma-1 receptor, Mitochondria, Mitochondrion-associated ER membrane (MAM), Neurodegenerative disorders

## Abstract

The sigma-1 receptor (Sig-1R) is a chaperone that resides mainly at the mitochondrion-associated endoplasmic reticulum (ER) membrane (called the MAMs) and acts as a dynamic pluripotent modulator in living systems. At the MAM, the Sig-1R is known to play a role in regulating the Ca^2+^ signaling between ER and mitochondria and in maintaining the structural integrity of the MAM. The MAM serves as bridges between ER and mitochondria regulating multiple functions such as Ca^2+^ transfer, energy exchange, lipid synthesis and transports, and protein folding that are pivotal to cell survival and defense. Recently, emerging evidences indicate that the MAM is critical in maintaining neuronal homeostasis. Thus, given the specific localization of the Sig-1R at the MAM, we highlight and propose that the direct or indirect regulations of the Sig-1R on mitochondrial functions may relate to neurodegenerative diseases including Alzheimer’s disease (AD), Parkinson’s disease (PD), Huntington’s disease (HD) and amyotrophic lateral sclerosis (ALS). In addition, the promising use of Sig-1R ligands to rescue mitochondrial dysfunction-induced neurodegeneration is addressed.

## Background

The sigma-1 receptor (Sig-1R) is an endoplasmic reticulum (ER) chaperone protein located primarily at the mitochondrion-associated ER membrane (MAM) that plays a variety of important roles in the cell. One of the functions of the Sig-1R is to regulate Ca^2+^ signaling between the ER and mitochondria for example by coupling to ankyrin B and inositol 1,4,5-trisphosphate receptor (IP3R) [1]. Sig-1R acts in an agonist/antagonist-sensitive manner to coordinate the coupling of ankyrin B to type 3 IP3R (IP3R3) to control Ca^2+^ signaling. The signaling pathway between Sig-1Rs, IP3R3s, and Ca^2+^ was found to relate to cellular survival against ER stress. When facing ER stress, the Sig-1R dissociates from cognate co-chaperone BiP and acts as a free chaperone to stabilize IP3R3s to increase Ca^2+^ signaling from ER into mitochondria to facilitate the production of ATP [2]. The Sig-1R also regulates Ca^2+^ influx by attenuating the coupling of the ER Ca^2+^ sensor STIM1 to Orai1 [3]. Crottès et al. studied the relationship between Sig-1R and ion channels in cancer cells, they reported that cancer cells expressed active Sig-1Rs that modulated a variety of ion channel families [4]. Sig-1Rs effectively altered the cell’s electrical plasticity, allowing the cell to become better suited for survival in a cancerous environment. The Sig-1R has also been implicated as an ion channel regulator in amyotrophic lateral sclerosis (ALS), a neurodegenerative disease that affects motor neurons. It was recently shown that motor neurons have the highest levels of Sig-1Rs in the central nervous system (CNS), and that Sig-1Rs may help direct the flow of ions through potassium channels [5]. This would be a way of reducing the excitability of motor neurons, therefore slowing the progression of ALS.

As well related to the ALS example, the Sig-1R may involve in the development and maintenance of axons and neurons. Sig-1R-lipid interactions are important in both oligodendrocyte (OL) differentiation and axon extensions. Sig-1Rs target galactosylceramide (GalCer)- and cholesterol-enriched lipid microdomains on the ER of OLs, and may thus modulate myelination by controlling the dynamics of the lipid transport to the myelin membrane [6]. Recently, Tsai et al. reported that the Sig-1R can modulate tau phosphorylation and axon development through an association with myristic acid and the cdk 5 activator p35 [7, 8]. The Sig-1R binds myristic acid to facilitate the myristoylation of p35 and promote the p35 turnover which, as a result, reduces the available p25 which would otherwise over-activate cdk5 leading to the hyperphosphorylation of Tau and retardation of axon growth. Hippocampal dendritic spine formation is also regulated by Sig-1Rs. The redox state of neurons determines the activity of the ER-mitochondrion-TIAM1-Rac1 GTP signaling pathway that is a component of dendritic spine development. The Sig-1R plays a part in this process by scavenging free radicals that would otherwise cause oxidative stress at the beginning of the pathway and attenuate dendrite formation [9].

Dysregulation of axonal maintenance may cause neurodegenerative and psychiatric disorders, such as Alzheimer’s disease (AD), Parkinson’s disease (PD), and schizophrenia. It has been shown that functional Sig-1Rs can help mitigate symptoms of some neurodegenerative disorders, although they can also be involved in the establishment of certain other diseases [10]. For this reason, Sig-1R ligands, both agonists and antagonists, are of great interest as potential therapeutic agents against CNS disorders.

The Sig-1R has also been shown to help protect cells from mitochondria-derived reactive oxidative species (ROS) associated damages. IRE1 is one of three ER stress sensors specifically located at the MAM to respond to stress caused by mitochondria or ER-derived ROS [11]. Upon ER stress, IRE1 undergoes dimerization and phosphorylation leading to its active endonuclease form. IRE1 then splices XBP1 mRNA with the end result being an upregulation of ER chaperones that can help mitigate stress. The Sig-1R mediates this process by stabilizing IRE1 during its activation.

The Sig-1R has an important function in regulating gene transcription. It was discovered that the Sig-1R, which normally localizes at the ER, can translocate to the nuclear envelope where it binds emerin that in turn recruits barrier-to-autointegration factor (BAF) and histone deacetylase (HDAC) to form a complex with specific protein 3 (Sp3) which can then suppress the gene transcription of monoamine oxidase B (MAOB) [12].

Thus, the Sig-1R plays a role in the mediation of many cellular functions, making it a protein of great interest for treatments of neurological disorders.

## Sig-1R regulates mitochondrial functions

Mitochondria are intracellular “powerhouse” organelles responsible for certain biogenesis and fundamental cellular energy processes [13]. Unlike other organelles in the cell, they are pretty much functionally autonomous since mitochondria have their own set of genomes mitochondrial DNA (mtDNA) [14], and can generate cellular energy. Most scientists prefer the endosymbiotic theories that the mitochondrial origin traces back to 1.5 billion years ago, arising from the endosymbiotic α-proteobacteria, in which free-living proteobacteria were taken inside another cell to form an endosymbiont and later evolved into an organelle [15]. Mitochondria contain multiple membrane compartments like their ancestors, including outer membrane, intermembrane space, inner membrane, boundary membrane, cristae and matrix [16]. Mitochondrion is also a dynamic organelle with constitutive fission, fusion, and is able to migrate or undergo mitophagy for manipulating the population of mitochondria and maintaining the metabolic homeostasis in different metabolic states [17, 18].

Mitochondrion is noted as a main source of ATP through oxidative phosphorylation that takes place in the inner membrane, comprising a series of respiratory chain complexes work cooperatively to drive the ATP production [16]. Apart from this, other metabolic process such as citric acid cycle (TCA cycle or Krebs cycle), synthesis of the heme groups and β-oxidation of fatty acids all occur in mitochondria [19]. Mitochondria also play important role in the Ca^2+^ signaling [20], production of ROS [21] and cellular apoptosis [22]. Therefore, mutation of the genes in mtDNA or nuclear genes coding for the metabolic process as well as dysfunction of some direct or indirect regulations of the mitochondrial proteins can lead to mitochondrial dysfunctions, causing multiple symptoms and diseases [23, 24].

The discovery of MAM dated back in the late 1950s when the association between ER and mitochondria was first identified by the electron microscopic examination in fish gills [25]. Subsequent studies with a sequel of improved protocols led to the isolation and characterization of biochemically distinct domains of ER-interacting mitochondria [26, 27]. To date, it is generally recognized that ER and mitochondria form contact sites via proteins that tether ER and mitochondrial membranes [28, 29]. These microdomains at ER-mitochondria junctions govern diverse cellular functions such as Ca^2+^ transfer, energy exchange, lipid synthesis and transports, and protein folding that are pivotal to cell survival and defense. Residing at the ER-mitochondra contact sites, Sig-1Rs not only regulate ER Ca^2+^ levels and protein degradations, they also govern cellular activities that take place within that specific MAM domain. Therefore, the Sig-1Rs serve as a communicator that bridges these two organelles and plays pivotal roles in mitochondrial functions. The Sig-1R and the mitochondrion both play multiple roles in the cell. Mitochondria are the main regulator of cell survival/death as well as that for the ROS production. How Sig-1Rs exert their cellular activities through direct or indirect regulations of mitochondrial functions will be described and/or proposed as follows.

### Maintains mitochondrial integrity

**Fig. 1:**
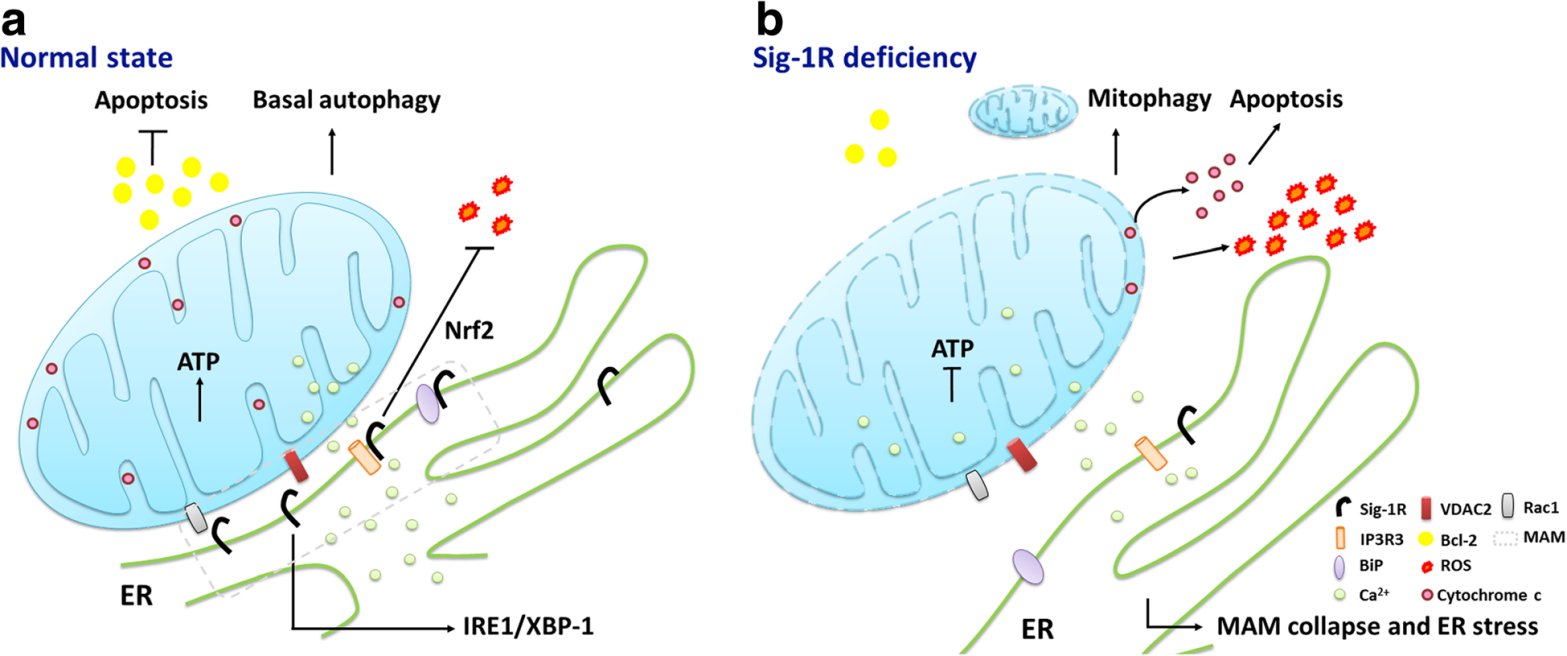
The putative model of Sig-1R at the MAM. **a** In the normal state, the Sig-1R interacts with BiP. Upon agonist stimulation or the decline of ER Ca^2+^, the Sig-1R dissociates from BiP to stabilize the IP3R3 for Ca^2+^ signaling from ER into mitochondria and to regulate the IRE1-dependent pathway to counteract the ER stress. The Sig-1R protects against apoptosis and ROS via the Bcl-2- or Nrf2-dependent pathways. Sig-1Rs can also associate with VDAC2 or Rac1. **b** The depletion of Sig-1R leads to an abnormal Ca^2+^ signaling between ER and mitochondria and the disruption of the ATP production. Enhanced ROS production, increased cytochrome c release, or reduced Bcl-2 can cause ER stress that leads to the collapse of the MAM and consequently enhanced autophagy or apoptotic cell death

Microdomain of high Ca^2+^ ion concentration is transiently generated in proximity to IP3 (inositol 1,4,5-trisphosphate)-sensitive channels and is surveyed by nearby mitochondria [30–32]. This microdomain for efficient Ca^2+^ transfer is called mitochondrial associated ER membrane (the MAM) [33, 34]. Ca^2+^ ion releasing from ER into the mitochondrial matrix can affect mitochondrial functions including the activation of metabolic enzymes for ATP production and the promotion of apoptosis cascades [35]. In the resting state, Sig-1Rs form a complex with the chaperone BiP at the MAM (Fig. 1a). Upon the ER Ca^2+^ depletion or the Sig-1R agonist stimulation, Sig-1Rs dissociate from BiP to chaperone IP3R3s, leading to a prolonged Ca^2+^ transfer from ER into mitochondria. Sig-1Rs can also translocate from the MAM to the entire ER network under continuously low ER Ca^2+^ concentration such as that caused by ER stress [2]. A splice variant of Sig-1R which lacks 47 ribonucleotides encoding for exon 2 forms a complex with Sig-1R but not with IP3R in the MAM. Therefore, overexpression of this variant interferes with normal Sig-1R functions such as the mitochondrial IP3R-mediated Ca^2+^ uptake. The Sig-1R variant also suppresses mitochondrial ATP production following ER stress, thus enhancing cellular apoptosis [36]. Overexpression of another Sig-1R variant, E102Q, impairs mitochondrial ATP production and elicits neuronal cell death [37]. These findings indicate that the Sig-1R regulates mitochondrial homeostasis, and some of the Sig-1R-interacting proteins may reside in the mitochondria. Using immunoprecipitation assay, Sig-1R was found to interact with mitochondrial Rac1 which is a critical regulator for neurogenesis, and formed complexes with IP3R and Bcl-2 in isolated mitochondria [38]. The Sig-1R agonist (+)-pentazocine further increased this interaction while the antagonist haloperidol cannot. (+)-Pentazocine also led to the phosphorylation of Bad and the NADPH-dependent production of ROS, suggesting that Sig-1R might act through the Rac1 signaling to induce mild oxidative stress and cell survival pathways. The roles of Sig-1Rs on restoring Ca^2+^ transferring into mitochondria, ATP productions, and mitochondrial morphology have also been demonstrated in the Sig-1R agonist SA4503-treated cardiomyocytes [39]. Consequently, Sig-1Rs play an important role in maintaining mitochondrial integrity as the aberrant neuronal mitochondrial aggregates or fragments have been associated with Sig-1R deficiency. Silencing of Sig-1Rs in hippocampal neurons leads to shorter and smaller mitochondria as well as aberrant mitochondria membrane potentials [9].

### Improves cell survival and stress response via mitochondria

Mitochondrial metabolism is closely related to several of the critical cellular functions including survival or apoptosis. The mitochondrial Ca^2+^ surge from the ER causes a mitochondrial Ca^2+^ overload, thus stimulating mitochondria to release multiple apoptotic factors such as cytochrome c that in turn activates caspase and leads to apoptosis [35]. The anti-apoptotic Bcl-2 family plays crucial roles in determining cellular survivals against apoptotic pathway [40]. It was found that the Sig-1R promoted cellular survival by regulating the Bcl-2 which at least in part exists on mitochondria, while silencing of Sig-1Rs down-regulated Bcl-2 mRNA expression and the effects were rescued by ROS scavengers or the inhibitor of the ROS-inducible transcription factor nuclear factor κB (NF-κB). Silencing of Sig-1Rs also enhanced hydrogen peroxide (H_2_O_2_)-induced cell apoptosis [41]. Sig-1R agonists protected neurons against the insults caused by oxygen–glucose deprivation or glutamate stimulation through the Bcl-2 pathway [42]. The transcriptome analysis revealed that Bcl-2 levels decreased in the Sig-1R KO retina [43]. In addition to the Bcl-2 family, Sig-1Rs protected retinal ganglion cell against glutamate-induced cell apoptosis by regulating Ca^2+^ signaling and inhibiting the activation of pro-apoptotic factors such as Bax and caspase-3 [44]. Conversely, knocking down Sig-1Rs in neurons caused a decrease of mitochondrial membrane potential and the release of cytochrome c, leading to disrupted cytoskeleton networks and the consequential immature formation of dendritic spines [9]. The Sig-1R is also involved in the pro-apoptotic pathways. Sig-1R ligands haven been shown to induce tumor cell death through the activation of caspase cascades, Ca^2+^-dependent activation of phospholipase C (PLC), Ca^2+^-independent inhibition of PI3K signaling [45], or the HIF-1α pathway [46]. Methamphetamine induced microglia apoptosis by activation of the MAPK, PI3K/Akt and p53 pathways, while blockage of the Sig-1R suppressed pro-apoptotic factors such as Bax, caspase-3 and caspase-9 induced by methamphetamine [47].

ER stress stimulates cells to activate the unfolded protein response (UPR) to cope with the stress resulting from accumulation of unfolded proteins in the ER [48]. Early phases of ER stress trigger an increase in mitochondrial ATP levels and oxygen consumption which depend critically on the ER-mitochondrion coupling and Ca^2+^ transfer from ER into mitochondria [49, 50], implying the metabolic regulation of mitochondria by the ER. The three major sensors of the UPR are PERK, IRE1 and ATF6 [48]. Sig-1Rs stabilized IRE1 at the MAM when cells were under ER stress. Deficiency of Sig-1R caused cell apoptosis by compromising the IRE1-XBP1 signaling. Treatment of cells with mitochondrial ROS inducer, antimycin A, showed that the mitochondrial-derived ROS triggered the IRE1-XBP1 signaling but not the ATF6 or PERK signaling pathway toward Sig-1Rs [11]. It was suggested that the mRNA of ATF6 showed profound changes in the retinal Müller glial cells isolated from Sig-1R KO mice [43], and microarray analyses revealed that silencing of Sig-1Rs influenced the expression of genes related to the ER pathway in primary hippocampal neurons [51]. Ligand such as (+)-pentazocine could attenuate the mRNA level of ER stress proteins PERK, ATF4, ATF6, IRE1, and CHOP that were upregulated in retinal ganglion cells exposed to oxidative stress [52]. ATF4 also interacted with the 5′ flanking region of *SIGMAR1*, and transcriptionally regulated the Sig-1R in the PERK/eIF2α/ATF4 pathway under ER stress [53], moreover, fluvoxamine, a selective serotonin reuptake inhibitor with affinity for Sig-1R, induced Sig-1R expression involving ATF4 without invoking the PERK pathway [54].

### Regulates oxidative stress derived from mitochondria

Free radicals play pivotal biological roles in cells including signal transduction, gene transcription and enzymatic activity regulation. However, unbalanced ROS productions in neuronal microenvironments caused free radical-induced lipid and protein modifications and DNA damages, generated many byproducts that are harmful to the cells, and led to manifestation of neurodegenerative diseases [55]. The mitochondrion is one of the main sources that produces oxidants in cells via consumption of O_2_ in the aerobic respiration [55]. One might wonder how the Sig-1R counterbalances the excess ROS. The Sig-1R has been reported to regulate oxidative stress responses and involve thus in the regulation of neuroplasticity through Rac1 GTPase. Paradoxically however, treatment of bovine brain mitochondria with Sig-1R agonist (+)-pentazocine led to the NADPH-dependent production of ROS [38]. Activation of Sig-1Rs through agonists has been reported to mitigate cellular stress. For example, the Sig-1R agonist blocked lipid peroxidation in β-amyloid (Aβ) peptide-injected mice [56], reduced nitrosative and oxidative stress on proteins after traumatic brain injury (TBI) [57], and mitigated the oxidative stress-induced cell death in human lens cell line [58]. These observations implicate the involvement of Sig-1Rs in neuroprotection. Emerging evidence provides insights to the underlying mechanisms of oxidative insults mediated by Sig-1Rs. A report showed that higher levels of ROS were observed in the livers, lungs and hepatocytes of Sig-1R KO mice when compared to that from the WT mice, suggesting that the KO mice were under oxidative stress. Antioxidant protein peroxiredoxin 6 (Prdx6) and the ER chaperone BiP were also increased in Sig-1R KO animals. Further analysis revealed that Sig-1R may upregulate NADPH quinone oxidoreductase 1 (NQO1) and SOD1 mRNA expression through antioxidant response element (ARE) [59]. The transcription factor Nrf2 (nuclear factor erythroid 2-related factor 2) binds to the ARE and regulates genes that are involved in cellular protection against oxidative stress-induced cell death [60]. Silencing of Sig-1Rs in primary hippocampal neurons also induced expression of genes related to the Nrf2-mediated oxidative stress pathway as shown from a microarray analysis [51]. Additionally, in a cellular model using Sig-1R KO Müller glia cells, the ROS levels were increased in KO cells with a concomitant reduced level of Nrf2 and the resultant Nrf2-ARE binding affinity [61]. Several genes involved in the mitochondrial metabolic process are transcriptionally regulated by Nrf2; therefore, Nrf2 also affects mitochondrial functions such as mitochondrial membrane potential, ATP synthesis, and mitochondrial fatty acid oxidation [62]. Although Nrf2 is considered as a transcription factor, it has been proposed that Nrf2 protects mitochondria from oxidant stress possibly through direct interaction with the mitochondrial outer membrane [63]. Moreover, a zinc finger protein 179 that has been identified as a Sig-1R downstream effector, exhibiting a neuroprotective role in the H_2_O_2_-induced ROS insult model [64]. The exact interactive connections between Sig-1R, Nrf2 and mitochondria as well as other neuroprotective mechanisms of Sig-1Rs in combating ROS remain to be totally clarified.

### Regulates autophagy via mitochondria

Autophagy is triggered upon cells are under stress such as nutrient starvation, ER stress, and pathogen infection. It is the process that cells strive for survival by invoking self-degradation of cellular components in which double-membrane autophagosomes engulf protein aggregates, organelles, portions of cytoplasm and fuse with lysosomes for energy demand [65]. Oxidative stress damages mitochondria while mitochondrion itself is also a substrate of autophagy, namely, mitophagy [66]. There are molecules that may provide link of autophagy to the MAM including IP3R which its signaling is required to maintain the autophagy suppression. Lack of IP3R decreased mitochondrial Ca^2+^ uptake and activated autophagy in the AMPK pathway [35, 67]. Part of the mitophagy is initiated when PINK1 recruits Parkin that targets mitochondria, causing the ubiquitination of the mitochondrial outer membrane protein voltage-dependent anion channel 1 (VDAC1) that is further recognized by p62 for degradation [66, 68]. Moreover, it is also suggested that autophagy originates from the MAM where nucleation of the isolation membrane may occur [69]. Therefore, emerging evidences suggest the role of the Sig-1R in autophagy. The Sig-1R antagonist 1-(4-iodophenyl)-3-(2-adamantyl)guanidine (IPAG) or haloperidol stimulated UPR and autophagic flux that depended on Sig-1R in a time-lapsed manner. UPR induction preceded autophagosome formation, and inhibition of UPR or autophagy accelerated cellular apoptosis that induced by antagonizing Sig-1R activities [70]. Silencing or loss of Sig-1Rs led to widened ER morphology, dissolution of mitochondrial cristae structure, and enhanced mitophagy in cells that were accompanied with impaired fusion between autophagosome and lysosomes, lipid raft destabilization, and impaired endolysosomal pathways [71]. Leptomycin B and thapsigargin caused the sequestration of Sig-1R within the nucleus with a resultant partial co-localization with p62 which is an important mediator in the proteasome and autophagy degradation systems [72]. Silencing of Sig-1Rs or employing the Sig-1R antagonist also demonstrated that cocaine, a Sig-1R agonist, induced autophagy in astrocytes through the Sig-1R mediated pathway [73]. Moreover, treatment of the Sig-1R antagonist increased the expression of the monosialotetrahexosylganglioside (GM1) and the accumulation of GM1 in the autophagosomes, demonstrating a relation between Sig-1R and gangliosides [74]. Interestingly, silencing of Sig-1Rs blocked autophagy at the isolation membrane expansion/LC3 lipidation stage [75], implicating the association of Sig-1R with the formation of autophpagy at the MAM as well as its ability to regulate cholesterol/lipid.

### Regulates lipid transport and steroidogenesis via mitochondria

It has been demonstrated that certain lipids are imported into mitochondria, for instance, phosphatidylserines are imported into mitochondria from the MAM contact sites to decarboxylate to phosphatidylethanolamine [29]. Sig-1Rs participate in the lipid synthesis and can bind simple sphingolipids such as ceramides [76]. MAM are enriched in cholesterol and sphingolipids, and form MAM-derived detergent-resistant membranes. Those detergent-resistant microdomains also regulate the anchoring of Sig-1R to the MAM. Sig-1Rs can interact with steroidogenic acute regulatory protein (StAR) and the voltage-dependent anion channel 2 (VDAC2) [77] which is a member of the mitochondrial porin family that transports metabolites across the mitochondrial outer membrane [78, 79]. At the MAM, VDAC2 regulates and interacts with StAR as a critical step to transport cholesterol into mitochondria for steroidogenesis [80]. Noteworthy, another study indicated that silencing of Sig-1Rs did not change the expression of ER and mitochondrial resident proteins but led to the reduced synthesis of pregnenolone. The interaction of the Sig-1R between VDAC2 and StAR, suggesting a role of Sig-1Rs in cholesterol trafficking and steroidogenesis at the MAM [77, 79]. Recently, it was also demonstrated that the Sig-1R can directly interact with myristic acid, promote p35 turnover, and regulate Tau phosphorylation and axon extension [7, 8]. The exact relation between Sig-1Rs and other lipids at the MAM remains to be clarified.

### Putative Sig-1R interacting proteins in mitochondria

Bioinformatics analyses identified several putative Sig-1R interacting proteins in mitochondria [81], including cytochrome C1 (CYC1), prohibitin (PHB), solute carrier family 25 member 11 (SLC25A11) and solute carrier family 25 member 39 (SLC25A39) [82]. Some of these proteins were demonstrated to be involved in the neurodegenerative disease or cellular protection. CYC1 is a subunit of mitochondrial complex III, playing roles in response to oxidative stress and the generation of superoxide anion in the mitochondrial respiratory chain [83, 84]. CYC1 is also identified as neuroglobin binding protein and the CYC1-neuroglobin association may be involved in the ATP production [83, 84]. Mitochondrial PHB families control cell proliferation, cristae morphogenesis and can regulate the fusion machinery of mitochondria [85]. SLC25 belongs to a family of transporters that functions in the shuttling of the metabolites across the inner mitochondrial membrane [86]. Inhibition of the SLC25A11 function decreased the mitochondrial GSH level in cerebellar astrocytes [87]. However, the direct demonstration of those proteins’ interactions with Sig-1Rs need to be investigated; so do the functional consequences of those interactions.

## Mitochondrial-associated neurological disorders and Sig-1R

Neurons and muscle cells contain high levels of mitochondria due to a high demand of energy. The CNS has a high rate of metabolism because neurons participate in facilitating the neurotransmission and extending axons and dendrites to neighboring cells for impulse transmission. Neurons exert plasticity, exhibiting complex morphologies, and constitutively undergo synaptic modulations when stimulated. Therefore, mitochondrial dysfunction can be detrimental to neurons [88] and has been extensively discussed in neurodegeneration [23, 89, 90]. Disruptions of the microdomains at ER-mitochondria contacts were found to relate to many neurological disorders [91–93]. Mechanisms involved in the progression of these diseases include dysfunction of mitochondria, imbalance of the Ca^2+^ homeostasis, ER stress, oxidative stress and autophagy. Stationed at the MAM, the Sig-1R acts as an intracellular organelle modulator between ER, mitochondria, nucleus, and the plasma membrane upon stimulations [82]. The Sig-1R is associated with many neurological disorders [94, 95], including AD [96], PD [97], ALS [5], HD [98], stroke/ischemia [99, 100], neuropathic pain [101], and certain psychiatric disorders [102]. Emerging evidences suggest that Sig-1R functions as an amplifier of intracellular signaling [95]. Sig-1R KO impaired neurogenesis in mice with depressive-like immobility phenotype [103–105]. Deficiency of Sig-1Rs aggravates the progression in many neurodegenerative models, while reinstating Sig-1Rs or agonistic activation restores neuronal functions and alleviates disease progression. How Sig-1Rs may regulate neurodegenerative diseases via a direct or indirect regulation on mitochondria, especially via the MAM, is described in the following sections.

### Sig-1R in AD

The major symptoms of AD include selective cognitive decline and memory loss, which are now accepted as being caused by the Aβ plaques and the tau neurofibrillary tangles. Aβ is generated from the serial enzymatic digestion of amyloid precursor protein (APP) which has been found to accumulate in the mitochondrial import channel in AD brains [106]. Aβ also accumulates in the mitochondria of AD patients and APP transgenic mouse [107], and is associated with elevated H_2_O_2_  and decreased cytochrome c oxidase activities in an animal model [108]. Aβ affects mitochondrial response to metabolic status by interacting with mitochondrial enzyme or disrupts synaptic functions by attenuating mitochondrial trafficking [109, 110]. Recently, it has been demonstrated that Aβ is generated intracellularly at MAM and may influence ER, mitochondrial and the MAM’s function [111]. Afobazole, a Sig-1R agonist, could lessen the increased Ca^2+^ caused by Aβ_25–35_ through the activation of Sig-1R. Afobazole reduced NO production, prevented upregulation of the proapoptotic protein Bax, activated caspase-3, and inhibited the downregulation of Bcl-2 induced by Aβ_25–35_ [112]. Up-regulation of Sig-1R was found in the APP_Swe/Lon_ mouse brain prior to the plaque formations, while decreased Sig-1R protein levels were observed in the human cortical postmortem brain tissue [113]. The Sig-1R expression is critical to the coupling of the ER-mitochondria contacts since the activation of Sig-1R in Aβ-treated cells significantly increased the Ca^2+^ shuttling from ER into mitochondria. Aβ also increased the expression of MAM-associated proteins such as IP3R3 and increased ER-mitochondria contacts in hippocampal neurons. Similar results were found in PET scan studies, in which Sig-1R expressions were lower in the brain of early AD patients [114]. On the other hand, the mitochondrial cholesterol influx was increased with concomitantly increased levels of Sig-1R and VDAC at MAMs in an old AD mouse model, indicating a relation of those MAM proteins in cholesterol trafficking [115]. Protein phosphatase 2A (PP2A) interacts with IP3R3 and Akt, and can regulate IP3R3 phosphorylation state [116]. In a brain endothelial cell culture model, okadaic acid-induced PP2A inhibition was accompanied by elevations of phosphorylated tau, ER stress markers, and Sig-1Rs as well as the Ca^2+^ overload in the mitochondria [117]. Brain vessels from 3xTg-AD mice also showed decreased PP2A. Apolipoprotein E (APOE) is another risk factor that is implicated in AD. The polymorphism analysis revealed that *SIGMAR1* and *APOE* may interact to influence the severity of AD [118]. Further, it was demonstrated that the ER–mitochondrion communication and the function of the MAM are increased significantly in cells treated with astrocyte conditioned medium containing APOE4 [119], suggesting a link to the Sig-1R. γ-Secretase complex is one of the enzymes that engages in the processing of APP to produce Aβ. The subunits of the γ-secretase complex, presenilin-1 (PS1) and presenilin-2 (PS2), have been found to localize at the MAM [120]. Increased MAM activity was detected in mouse embryonic fibroblasts lacking PS1 and PS2 [121]. Overexpression or down-regulation of PS2 caused the fluctuation of Ca^2+^ concentrations between ER and mitochondria [122]. In the tissues of an AD-associated mutant, PS1-E280A, the ER-mitochondrion tethering was impaired and voltage-gated P/Q-type Ca^2+^ channels, IP3Rs and Ca^2+^-dependent mitochondrial transport proteins were reduced as well. Overexpression of this mutant altered the ER-mitochondrion tethering and associated transport in the neuronal cell [123]. Tau proteins may be involved in the pathogenesis of AD through their detrimental effect on mitochondria [124, 125]. However, the association of tau and Sig-1R as well as the PS processing mechanism mediated by Sig-1R have yet to be established.

### Sig-1R in PD

Parkinson’s disease is a slowly progressing disorder, causing impaired motor functions such as bradykinesia or tremor, and other non-motor complications. The pathological characteristic of PD is the deposit of Lewy bodies composed of α-synuclein, ubiquitin and neurofilaments [126]. α-Synuclein [127, 128], Parkin, PINK1 [68, 129–131], DJ-1 [132–134] and LRRK2 [135] have been demonstrated to be closely linked to the mitochondrial-related PD pathogenesis.

Sig-1R expressions were lower in putamen of PD patients as demonstrated by PET studies [114]. Dopamine toxicity is involved in the etiology of PD. Dopamine activated NF-κB while Sig-1Rs counteracted and inhibited the proteasomal conversion/activation of NF-κB. Silencing of Sig-1Rs in combination with dopamine treatment caused a synergistic proteasomal conversion of NF-κB p105 to the active form of p50, which is known to down-regulate Bcl-2 at the transcriptional level. Dopamine caused apoptosis in Sig-1R knockdown cells and the effects could be reversed by overexpression of Bcl-2 [136]. Accumulation of α-synuclein impaired mitochondrial complex I activity, and caused the release of cytochrome c and the elevation of mitochondrial Ca^2+^, nitric oxide (NO) and ROS concentrations [127, 128]. Moreover, α-synuclein controls mitochondrial Ca^2+^ homeostasis by enhancing the ER-mitochondria associations [137] and was later found to exist at the MAM where it modulates the mitochondrial morphology [138]. Intriguingly, Pailluson et al. demonstrated a closer link between MAM and PD [139]. Vesicle-associated membrane protein-associated protein B (VAPB) is an ER-resident protein and protein tyrosine phosphatase interacting protein 51 (PTPIP51) is an outer mitochondrial membrane protein. Both proteins function as a bridge tethering the ER and mitochondria. Residing at the MAM, α-synuclein also interacts with VAPB but not PTPIP51. Silencing of α-synuclein does not alter ER-mitochondria associations, while overexpression of wild-type and familial PD mutant α-synuclein disrupts the tethering between VAPB and PTPIP51 to loosen the ER-mitochondria contacts. The actions of α-synuclein include the loss of MAM domain, disruption of Ca^2+^ transferring between the two organelles, and the inhibition of ATP production. Neither expression of WT/mutant nor silencing of α-synuclein changed the protein expression of Sig-1R, indicating that α-synuclein may not influence the translational level of Sig-1R [139]. However, it remains to be investigated if the α-synuclein-induced reduction of the ER-mitochondria associations may involve the Sig-1R. Parkin and PINK1 work cooperatively to regulate the homeostasis of mitochondria, such as mitochondrial fission/fusion machinery, the integrity of mitochondria or mitophagy [68, 129–131]. DJ-1 exerts its neuroprotection by regulating the function of mitochondria [134], and its mutation also caused a reduction in the level of ATP [140]. Parkin and DJ-1 can both alter the ER-mitochondria crosstalks and tethering [141, 142]. A close examination on the association between Sig-1R and those proteins may provide more insights in the future.

### Sig-1R in HD

HD is an inherited disorder in an autosomal dominant pattern due to an elongated CAG repeat in the Huntingtin (Htt) gene, *HTT*, and is clinically characterized by progressive retardation in motor, cognition and psychiatric states [143]. HD mutation is associated with mitochondrial dysfunction and apoptotic pathways. Inhibition of mitochondrial function via the complex II inhibitor 3-nitropropionic acid (3NP) recapitulates HD-like symptoms in animals [144]. Mitochondrial fractionation revealed that Htt is present in the mitochondrial outer membrane. Mutant Htt protein induced mitochondrial permeability transition (MPT) accompanied by a significant release of cytochrome c [145]. Overexpressing of Htt proteins with 74 or 138 polyglutamine repeats induced mitochondrial fragmentation under oxidative stress, in which Htt 74 also caused cell death, reduction in ATP levels, and interference on the dynamics of mitochondrial fusion/fission [146]. Further, Htt could interact with Drp1 which controls mitochondrial fission, elevates Drp1 enzyme activities, and induces abnormal dynamics and anterograde movements of mitochondria, thus leading to disruption of synaptic functions [147].

Expression of N-terminal Htt proteins with expanded polyglutamine activates ER stress, increases BiP protein expression, and causes cell death in neuronal cells. Compound that inhibits ER stress such as salubrinal could rescue the cell death and eliminate protein aggregations resulting from mutant Htt proteins [148]. A similar approach was also used to investigate the relation between Sig-1R and mutant Htt. Sig-1R expression is decreased in mutant Htt protein-expressing cells [98]. Treatment of the Sig-1R agonist PRE084 counteracted the effects caused by mutant Htt by increasing cellular antioxidants, reducing the ROS level, increasing NF-*κ*B-p65, and activating NF-*κ*B signaling without changing mitochondrial Ca^2+^ concentration. A partial co-localization of Sig-1R with aggregates of cytoplasmic mutant Htt was observed, indicating that the Sig-1R may play some unknown roles in the Htt aggregates such as being hijacked by the aggregates with a loss of its function. Similar results were observed in that Sig-1Rs translocated and colocalized with the mutant Htt in the nucleus [149]. Although mitochondrial Ca^2+^ levels were not affected by mutant Htt proteins in this model, another report indicated that the interaction of type I IP3R with BiP was reduced in the HD mouse model that was accompany by impaired Ca^2+^ releasing activity of type I IP3R [150]. Moreover, a Sig-1R ligand, pridopidine was found to improve motor function in a HD R6/2 mouse model. Pridopidine increased the expression of neuroprotective factors, such as BDNF and DARPP32, and reduced the size of Htt aggregates in HD mice. The effect of pridopidine was abolished in the presence of Sig-1R antagonist in cellular model, implying that the Sig-1R was involved in the neuroprotective functions of pridopidine [151]. Pridopidine activated neuronal plasticity and survival pathways, and the Sig-1R may represent a major regulator to increase the secretion of BDNF [152]. Further, in a YAC128 transgenic HD mouse model, it was demonstrated that pridopidine prevented the loss of medium spiny neurons through Sig-1R in aging YAC128 co-cultures. Pridopidine treatment also normalized the ER Ca^2+^ levels in medium spiny neurons in the co-culture system [153]. Although the MAM region has not been directly demonstrated to be involved in HD, the insightful information mentioned in this section implies a relation between Sig-1R’s function at MAM and HD may exist.

### Sig-1R in ALS

The clinical hallmark of ALS is the presence of upper and lower motor neuron dysfunction as seen in the limbs that can further manifested as muscular atrophy in other regions [154]. Mitochondrial pathology occurs as an initial event in a mouse model of ALS [155]. The motor nerve terminals from ALS patients contained abnormal Ca^2+^ concentrations and increased mitochondrial volumes [156]. Several risk factors have been identified in ALS and demonstrated to be involved in mitochondrial homeostasis, including SOD1 [157–159], FUS/TLS [160], TDP-43 [161], OPTN [162] and C9Orf72 [163]. SOD1 scavenges free superoxide radicals in the cells, and mutant SOD1 protein has been shown to bind to the cytoplasmic face of mitochondria [158]. SOD1 mutant mouse model demonstrated mitochondrial abnormalities, motor neuron death, and symptoms and pathology similar to those observed in ALS [157]. Motor neurons expressing mutant SOD1 also showed impairments in mitochondrial fusion in axons and soma, dysregulsation of mitochondrial retrograde axonal transport, and a reduction in the size of mitochondria [159].

Sig-1R proteins were reduced in the lumbar spinal cord of ALS. They were also accumulated in enlarged C-terminals and ER structures of alpha motor neurons. The disrupted Sig-1R localization was also observed in SOD1 transgenic mice [164]. A Sig-1R KO mouse model showed muscle weakness and motor neuron loss, and the inhibition of mitochondrial fission caused defect in mitochondrial axonal transport and axonal degeneration that were similar to that seen in Sig-1R deficiency samples. Those defects can be restored by the Ca^2+^ scavenging and ER stress inhibition in motor neurons [165]. The collapse of the MAM (Fig. 1b) was demonstrated as a common mechanism in Sig-1R- and SOD1-linked ALS models [166]. Watanabe et al. found that a homozygous mutation p.L95fs of *SIGMAR1* was identified in the inherited juvenile ALS. The mutant variant of Sig-1R showed reduced stability and was incapable of binding to IP3R3s. The mutant SOD1 was also detected at the MAM where the mutant was observed in neurons but not in astrocytes or other cell types of the SOD1 mouse model. Furthermore, deficiency of Sig-1Rs accelerated the onset of SOD-1-mediated ALS in mouse model. Deficiency of Sig-1R or accumulation of mutant SOD1 could induce the collapse of the MAM, leading to the mislocalization of IP3R3s, the activation of calpain, and the dysfunction of mitochondria. Administration of the Sig-1R agonist PRE-084 restored the Sig-1R-IP3R3 interaction and prevented the Sig-1R aggregation [166]. TDP-43 was found to form hyper-phosphorylated, ubiquitin-positive inclusions in ALS [167], and the ALS disease-associated mutant TDP-43 exhibited greater extent of mis-localization in mitochondria [161]. Moreover, pathologic TDP-43 that perturbs the ER-mitochondrion association was also observed [168]. The association of Sig-1R and TDP-43 was documented in a study in which a nonpolymorphic mutation in the 3′-untranslated region of *SIGMAR1* was identified in patients from the frontotemporal lobar degeneration-motor neuron disease (FTLD-MND) pedigree [169]. Brains of *SIGMAR1* mutation carriers showed cytoplasmic inclusions of TDP-43 or FUS. Overexpression of Sig-1R increased the mislocalization of TDP-43 and FUS from the nucleus to the cytoplasm while Sig-1R antagonists reduced the cytoplasmic to nuclear TDP-43 ratio. The mutation of the *SIGMAR1* (p.E102Q) has also been found in the ALS patients [170]. Overexpression of this mutant increased mitochondrial damage, induced autophagic cell death, and led to mislocalized TDP-43 [37, 171]. The Sig-1R was observed in the neuronal nuclear inclusions in various neurodegenerative diseases, suggesting that the Sig-1R might move laterally between the nucleus and the cytoplasm under certain conditions [72]. Those findings suggest a role of Sig-1R as well as the importance of MAM integrity in ALS.

### Sig-1R endogenous ligands in neurodegenerative diseases

In addition to the synthetic agonists and antagonists listed above, the endogenous ligands of Sig-1Rs include the steroids (progesterone, DHEA-sulfate and testosterone) [172, 173], hallucinogen N,N-dimethyltryptamine (DMT) [174], sphingosine [175, 176] and monoglycosylated-ceramide [76, 177]. Progesterone was found to regulate free radical metabolism in brain mitochondria and provides neuroprotective and anti-inflammatory effects in the CNS [178, 179]. A motor neuron degeneration mouse model showed less pronounced abnormal mitochondria morphologies after receiving progesterone [180], and progesterone also regulate AD-like neuropathologies in female 3xTg-AD mice [181]. Some steroids and progesterone are synthesized at specific location of ER, and progesterone can inhibit the dissociation of Sig-1R and BiP [2, 182]. On the contrary, pregnenolone sulfate also caused the dissociation of an ankyrin B isoform from IP3R3, eliciting Ca^2+^ concentration and signaling [1, 183]. DMT is a hallucinogen found in human brain and is postulated to generate endogenously under cellular stress [184]. Mice injected with DMT showed hypermobility, but the effects were not observed in the Sig-1R KO phenotype [174], indicating DMT binding to Sig-1R to modulate its actions. Therefore, a model has been proposed that low concentration of DMT dissociates Sig-1Rs from BiP, allowing Sig-1Rs to regulate IP3R3s at the MAM. The Ca^2+^ signaling increased from the ER into mitochondria as well as the ATP production while higher concentrations of DMT induced the translocation of Sig-1Rs from the MAM to other cellular compartments, and inhibited ion channel’s activities [185]. DMT producing enzyme also exhibited closed proximity to the Sig-1R in the motor neurons, implying the local synthesis of DMT in the wake of Sig-1R regulations [186]. Later studies showed that DMT mitigated hypoxic stress or modulated inflammatory responses via Sig-1R in iPSC-derived cortical neurons or immune cells [184, 187]. Sig-1Rs associate with simple sphingolipids such as ceramides [76] which regulate mitochondrial functions such as eliciting the release of proapoptotic factors from the mitochondria, ROS production from mitochondria, and lipid synthesis, and are also implicated in CNS pathologies [188, 189]. Identifying the putative endogenous ligands excludes the Sig-1R as an orphan receptor, and the later discovery on the chaperoning function via the IP3R3 re-defines the pivotal role of the Sig-1R, nevertheless, the subtle and coordinated actions/balances between Sig-1R and its putative endogenous ligands remain to be clarified to elucidate potential roles in the neurodegenerative diseases or other psychiatric illnesses toward Sig-1Rs.

## Conclusions and future perspective

The function of Sig-1R is activated when cells are under stress. The Sig-1R chaperone protein exerts pluripotent properties that can exist in the nuclear envelope, the nucleoplasmic reticulum, the MAM, the ER, and potentially the plasma membrane [190]. The main function of Sig-1R is to regulate the Ca^2+^ gradient between ER and mitochondria through the MAM. Recently, the crystal structure of Sig-1R proposing a trimeric architecture with a single transmembrane domain in each protomer, with one side facing the ER lumen and the other side facing the surface of the ER in cells [191]. This discovery will accelerate the pace in understanding the ligand binding state and other important cellular mechanisms of Sig-1R. The Sig-1R has been proven to play certain roles in many neurodegenerative diseases. Ligands of the Sig-1R have also been shown to exhibit neuroprotective properties, providing some potential promising therapies in the future. It has been proposed that many aggregated proteins related to neurodegenerative disease were imported into mitochondria [192]. The regulatory functions of the Sig-1R chaperone on mitochondria thus deserve thorough investigations. The MAM, thus Sig-1Rs, represents an important target in the treatment of neurodegenerative diseases (Fig. 1). Whether Sig-1R interactions with other MAM tethering proteins may relate to those diseases remains to be fully investigated.

## Abbreviations

3NP: 3-nitropropionic acid
AD: Alzheimer’s disease
ALS: Amyotrophic lateral sclerosis
APOE: Apolipoprotein E
APP: Amyloid precursor protein
ARE: Antioxidant response element
Aβ: β-amyloid
BAF: Barrier-to-autointegration factor
CNS: Central nervous system
CYC1: Cytochrome C1
DMT: N,N-dimethyltryptamine
ER: Endoplasmic reticulum
FTLD-MND: Frontotemporal lobar degeneration-motor neuron disease
GalCer: Galactosylceramide
GM1: Monosialotetrahexosylganglioside
H_2_O_2_: Hydrogen peroxide
HD: Huntington’s disease
HDAC: Histone deacetylase
Htt: Huntingtin
IP3: Inositol 1,4,5-trisphosphate
IP3R: Inositol 1,4,5-trisphosphate receptor
IP3R3: Type 3 inositol 1,4,5-trisphosphate receptor
IPAG: 1-(4-iodophenyl)-3-(2-adamantyl)guanidine
MAM: Mitochondrion-associated ER membrane
MAOB: Monoamine oxidase B
MPT: Mitochondrial permeability transition
mtDNA: mitochondrial DNA
NF-κB: Nuclear factor κB
NO: Nitric oxide
NQO1: NADPH quinone oxidoreductase 1
Nrf2: Nuclear factor erythroid 2-related factor 2
OL: Oligodendrocyte
PD: Parkinson’s disease
PHB: Prohibitin
PLC: Phospholipase C
PP2A: Protein phosphatase 2A
Prdx6: Peroxiredoxin 6
PS1: Presenilin-1
PS2: Presenilin-2
PTPIP51: Protein tyrosine phosphatase interacting protein 51
ROS: Reactive oxidative species
Sig-1R: Sigma-1 receptor
SLC25A11: Solute carrier family 25 member 11
SLC25A39: Solute carrier family 25 member 39
Sp3: Pecific protein 3
StAR: Steroidogenic acute regulatory protein
TBI: Traumatic brain injury
UPR: Unfolded protein response
VAPB: Vesicle-associated membrane protein-associated protein B
VDAC: Voltage-dependent anion channel
VDAC1: Voltage-dependent anion channel 1
VDAC2: Voltage-dependent anion channel 2
